# Structured diagnostic scheme clinical experience sharing: a prospective study of 320 cases of fever of unknown origin in a tertiary hospital in North China

**DOI:** 10.1186/s12879-023-08436-0

**Published:** 2023-07-07

**Authors:** Lin Jiang, Han Wu, Sen Zhao, Yu Zhang, Ning Song

**Affiliations:** 1 Department of Infectious Diseases, The Second Hospital of Hebei Medical University, Shijiazhuang, China; 2 Department of Critical Care Medicine, Shiyan Renmin Hospital, Shiyan, China

**Keywords:** Fevers of unknown origin, Cause, Prognosis

## Abstract

**Background:**

There has been little research on the long-term clinical outcomes of patients discharged due to undiagnosed fevers of unknown origin (FUO). The purpose of this study was to determine how fever of unknown origin (FUO) evolves over time and to determine the prognosis of patients in order to guide clinical diagnosis and treatment decisions.

**Methods:**

Based on FUO structured diagnosis scheme, prospectively included 320 patients who hospitalized at the Department of Infectious Diseases of the Second Hospital of Hebei Medical University from March 15, 2016 to December 31,2019 with FUO, to analysis the cause of FUO, pathogenetic distribution and prognosis, and to compare the etiological distribution of FUO between different years, genders, ages, and duration of fever.

**Results:**

Among the 320 patients, 279 were finally diagnosed through various types of examination or diagnostic methods, and the diagnosis rate was 87.2%. Among all the causes of FUO, 69.3% were infectious diseases, of which Urinary tract infection 12.8% and lung infection 9.7% were the most common. The majority of pathogens are bacteria. Among contagious diseases, brucellosis is the most common. Non-infectious inflammatory diseases were responsible for 6.3% of cases, of which systemic lupus erythematosus(SLE) 1.9% was the most common; 5% were neoplastic diseases; 5.3% were other diseases; and in 12.8% of cases, the cause was unclear. In 2018–2019, the proportion of infectious diseases in FUO was higher than 2016–2017 (*P* < 0.05). The proportion of infectious diseases was higher in men and older FUO than in women and young and middle-aged (*P* < 0.05). According to follow-up, the mortality rate of FUO patients during hospitalization was low at 1.9%.

**Conclusions:**

Infectious diseases are the principal cause of FUO. There are temporal differences in the etiological distribution of FUO, and the etiology of FUO is closely related to the prognosis. It is important to identify the etiology of patients with worsening or unrelieved disease.

**Supplementary Information:**

The online version contains supplementary material available at 10.1186/s12879-023-08436-0.

## Introduction

Petersdorf and Beeson formally proposed the definition of classic fever of unknown origin (FUO) in 1961 by observing and summarizing a series of patients with unexplained fever as follows: a temperature > 38.3 °C on several occasions over a period of more than 3 weeks without a diagnosis despite 1 week of inpatient investigation [1]. Currently, the aetiological classification of FUO includes infectious diseases, non-infectious inflammatory diseases, tumour diseases, other diseases and unknown diagnoses [2]. There are over 200 causes of FUO [3], it remains a difficult problem in clinical diagnosis.

The etiological distribution of FUO varies according to the study period and region [4–7], and the constituent ratio of infectious diseases in FUO etiology ranges from 23.1% to 68.34% [8, 9]. Notably, some studies have shown that contagious diseases are more common among infectious diseases, e.g., tuberculosis, brucellosis, typhoid fever [8, 10–12]. Therefore, early diagnosis of infectious diseases not only enables patients to receive timely and reasonable treatment, but also reduces the risk of major adverse public health events. Although there have been great advances in diagnostic techniques such as imaging techniques and laboratory tests since the definition of FUO was proposed, the unconfirmed rate is still as high as 8.35% to 53% due to the wide variety of FUO causes, atypical clinical manifestations, and the lack of specific diagnostic tools. Only a few studies to date have reported the outcome of patients who are discharged with undiagnosed FUO. Most undiagnosed FUO eventually fails to confirm the diagnosis, but patients mostly heal spontaneously, with a mortality rate of 6.9 to 18.6% [13–15]. It is crucial to conduct real-time dynamic studies on the etiology and prognosis of FUO.

In the early stage, Jia Weihua [16] is conducting a prospective study on 102 FUO patients, although it has short observational time and a small number of samples. This study is an extended study on the early stage study and further expands the sample size. At the same time, according to the latest diagnosis and treatment guidelines of FUO, we included computed tomography(CT), 2-[18F]-fluoro-2-deoxy-D-glucose(18F-FDG) -Positron Emission Tomography/Computed Tomography(PET/CT) (Abbreviate: 18F-FDG PET/CT), hemophagocytic syndrome-related examinations and other examination strategies in the etiological diagnosis strategy of FUO, and followed up all FUO patients to obtain some new data, which were summarized in this paper in order to improve clinicians' understanding of this disease.

## Methods

### Patients

This prospective study assessed patients aged ≥ 14 years with classic FUO from the Department of Infectious Diseases of the Second Hospital of Hebei Medical University between March 15, 2016 and December 31,2019. Classic FUO was diagnosed in patients meeting all of the following criteria based on the 1961 criteria [1], expert study consensus in China [17] and current definition of FUO.

- temperature ≥ 38.3 °C (101°F) on at least two occasions
- duration of illness ≥ 3 weeks or multiple febrile episodes in ≥ 3 weeks
- not immunocompromised (neutropenia for ≥ 1 week in the 3 months prior to the start of the fever; known HIV-infection; known hypogammaglobulinemia or use of 10 mg prednisone or equivalent for ≥ 2 weeks in the 3 months prior to the start of the fever)
- Diagnosis uncertain despite thorough history-taking, physical examination and the following investigations: erythrocyte sedimentation rate or C-reactive protein, haemoglobin, platelet count, leukocyte count and differentiation, electrolytes, creatinine, total protein, protein electrophoresis, alkaline phosphatase, aspartate aminotransferase, alanine aminotransferase, lactate dehydrogenase, creatine kinase, antinuclear antibodies, rheumatoid factor, microscopic urinalysis, ferritin, three blood cultures, urine culture, chest X-ray, abdominal ultrasonography and tuberculin skin test [18].

### Diagnostic workup

**Fig. 1:**
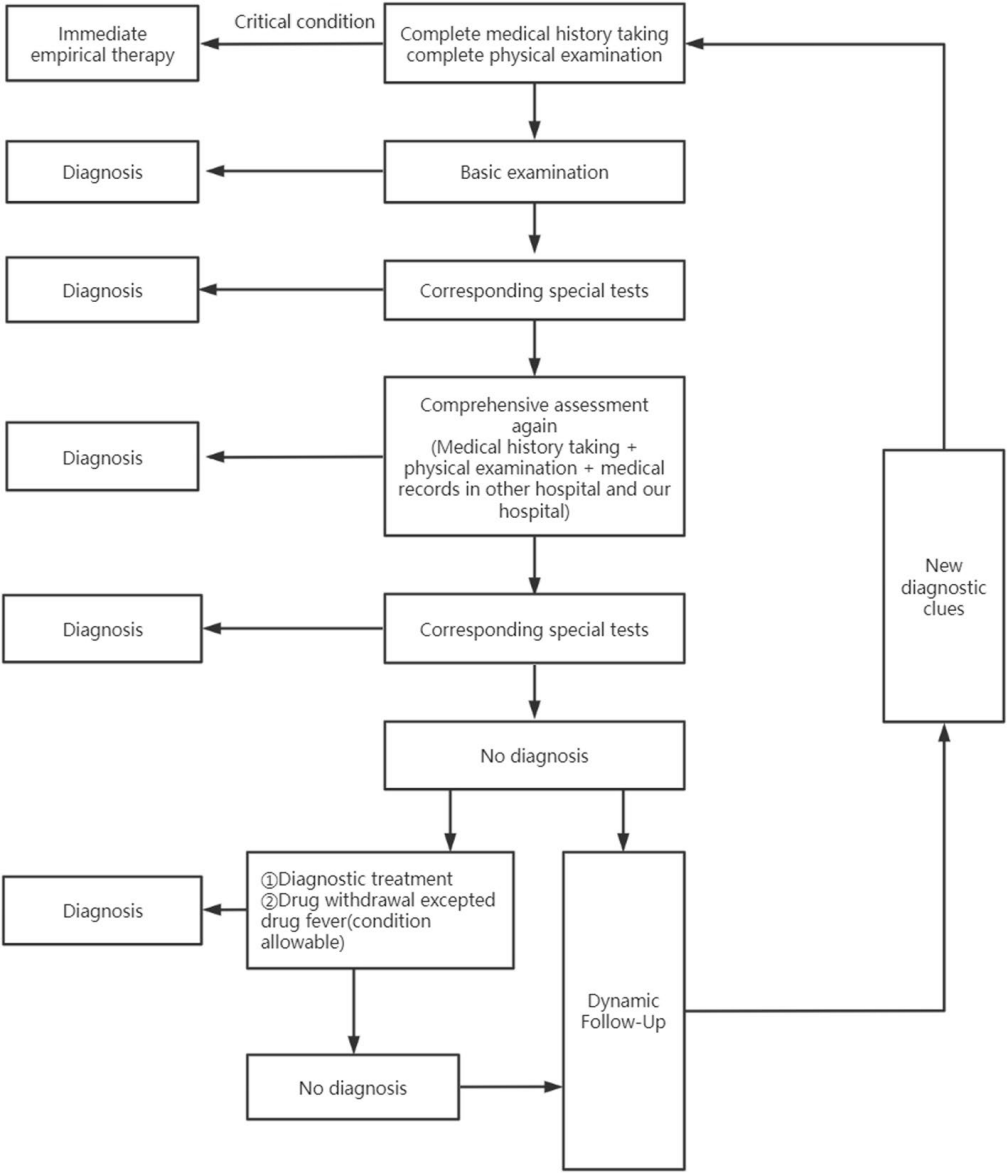
FUO structured diagnostic protocol (etiological distribution and diagnostic strategy study in 102 patients with fever of unknown origin adapted from Weihua Jia [16]). Basic examination, Special examination Listed below, (see Table 1)

In this study, a FUO research team consisting of experts from various disciplines including infectious diseases and respiratory medicine was established to develop a diagnostic protocol. After obtaining informed consent, in all patients, a structured diagnostic protocol was used (Fig. 1).The structured diagnostic protocol includes: ①A detailed medical history acquisition and physical examination to obtain preliminary diagnostic clues. Diagnostic clues are defined as all localizing signs, symptoms, and abnormalities potentially pointing toward a diagnosis. At the same time, for critical patients, empirical treatment immediately. ②Improves the basic checks to provide new. ③Made a diagnosis combined with medical history, physical examination, and basic examination clues. For patients with unknown diagnosis, further specific tests were performed. ④After the above examination was completed, the diagnosis cannot be clear, then the detailed medical history collection and physical examination were conducted again, and the auxiliary examination data including the external examination results were comprehensively analyzed. ⑤ If a new diagnostic clues appear, the relevant special examination is perfected again. ⑥ For FUO patients who cannot be diagnosed after multiple evaluation, give diagnostic treatment [19] after careful consideration, including empirical use of antibiotics, diagnostic anti-tuberculosis treatment, etc., drug withdrawal observation except for drug fever, and continuously observe the changes in the condition. ⑦ For FUO patients with mild clinical manifestations and stable condition, and the etiology is still unclear after comprehensive examination, long-term follow-up can be selected, and the etiology can be evaluated again when new diagnostic clues appear. (See Table 1 for the auxiliary inspection items).

**Table 1:** Auxiliary Inspection Items in the FUO Diagnostic Scheme

I. Basic examination: Hematuria routine, inflammatory indicators (erythrocyte sedimentation rate, C-reactive protein, procalcitonin), biochemical tests (myocardial enzymes, liver function, renal function, electrolytes, blood glucose, blood lipids), preoperative four items, 1,3-β-D glucan detection, tuberculosis series examination, virus series antibodies, respiratory pathogen series antibodies, tumor markers, rheumatism immune series, thyroid function, cardiac ultrasound, abdominal ultrasound, chest CT, abdominal CT, pelvic CT and other examinations
II. Special examination: Vascular ultrasound, blood, urine, sputum and other body fluid culture, soluble CD25 level in peripheral blood, naturalkiller (NK) cell activity detection, cerebrospinal fluid examination, bone marrow smear, bone marrow culture, bone marrow pathology, lymph node biopsy, skin, muscle, liver, large artery and other tissue and organ biopsy, 18F-FDG PET/CT examination, etc

### The tests by which a type of infection was confirmed

Laboratory diagnoses of infectious diseases are referred to the diagnostic criteria of the relevant diseases.Purified protein derivative (PPD) and interferon-γ release assay (IGRA) were adopted to diagnose pulmonary TB;Widal reaction were adopted to diagnose Typhoid. Tiger Red Plate Agglutination Test and Test-tube agglutination test were adopted to diagnose Brucellosis.

### Blood culture strategy

When blood culture was performed, a total of 2 bottles of 8—10 ml of blood were taken by the nurse for aerobic and anaerobic cultures respectively after strict aseptic operation.Bacterial identification use professional clinical bacterial identification methods such as the VITEK2 system, Phoenix100 system, or manually.Bacterial drug sensitivity testing was performed by automated instrumental method or paper diffusion method, and the results of drug sensitivity testing were determined according to the American Society for Clinical and Laboratory Standards 2016 edition standards.

### Etiological and follow-up

FUO etiology is divided into 4 categories [2], in addition, etiologies containing both of the five categories are called "multiple etiologies" [9]. The differences in the etiological distribution of FUO between the groups were compared according to year, gender, age (young: ≤ 44 years, middle-aged: 45 – 59 years, old: ≥ 60 years), and fever duration. All discharged FUO patients were followed up regularly by telephone to document survival/death until 30 June 2020.

### Statistical analysis

Statistical analyses were performed using IBM SPSS Statistics for Windows, V.22.0. Measurement data conforming to normal distribution are expressed as mean ± standard deviation, while those not conforming to normal distribution are expressed as median and interquartile range M (Q25, Q75). For measurement data conforming to normal distribution, group t test was used for comparison between two groups, and Wilcoxon rank sum test was used for those not conforming to normal distribution. Enumeration data were analyzed by chi-square test. Differences were considered statistically significant at *P* < 0.05.

## Results

### Patient characteristics

From March 15, 2016 to December 31, 2019, a total of 4320 inpatients were admitted to the Department of Infectious Diseases of the Second Hospital of Hebei Medical University, and a total of 320 (7.4%) met FUO criteria. Of 320 FUOs, 159 (49.7%) were male and 161 (50.3%) were female. Median age was 53 (35,66) years. The median fever duration was 32 (25, 60.75) days. Median follow-up was 18 (8.5, 28.5) months.

### Distribution of FUO etiology

Among 320 FUO patients, infectious diseases were the most common cause (*n* = 222, 69.3%), and the top two infected parts were urinary tract infections (*n* = 41, 12.8%) and pulmonary infections (*n* = 31, 9.7%). There were 20 cases (6.3%) of non-infectious inflammatory diseases, with systemic lupus erythematosus(SLE) being the most common (*n* = 6, 1.9%). Other diseases were found in 17 patients (5.3%), including necrotizing lymphadenitis, subacute thyroiditis in 8 patients each, and drug fever in 1 patient. Sixteen patients (5%) had neoplastic disease, mainly hematologic malignancies (*n* = 13, 4.0%), and the most common was lymphoma (*n* = 11, 3.4%). There were 4 cases (1.3%) with multiple etiologies: 1 case each of pneumonia combined with esophageal cancer and pneumonia combined with subacute thyroiditis, 1 case of necrotizing lymphadenitis combined with urinary tract infection, and 1 case of necrotizing lymphadenitis combined with viral meningitis. Undiagnosed 41 cases (12.8%). (See Table 2.) (At the end of the document text file.) Then describe the exact proportion of FUO etiology-wise with time intervals, according to mean follow-up time. (See Table 3.)

**Table 2:** Distribution of FUO etiology

Causes	Number of examples	Scale (%)
**Infectious Diseases**	**222**	**69.3**
** Single-site infection**	**110**	**34.3**
Urinary Department of Urology Infection	41	12.8
Pneumonia:	31	9.7
Blood flow infection	11	3.4
Hepatic abscess	8	2.5
Cholecystitis	2	0.6
Infectious endocarditis	8	2.5
Skin soft-tissue infection	2	0.6
Central nervous system infection	3	0.9
Adrenal abscess	1	0.3
Periappendicular abscess	1	0.3
Pulmonary Tuberculosis	1	0.3
Spinal tuberculosis	1	0.3
** Multisite mixed-infection**	**55**	**17.2**
Urology system infection + pneumonia	30	9.4
Urology system infection + cholecystitis	1	0.3
Urology system infection + blood flow infection	4	1.3
Urology system infection + skin soft-tissue infection	2	0.6
Pneumonia + cholecystitis	2	0.6
Pneumonia + blood flow Infection	2	0.6
Pneumonia + skin soft-tissue infection	1	0.3
Pneumonia + infective endocarditis	3	0.9
Pneumonia + Central nervous System Infection	2	0.6
Liver abscess, + blood flow infection	2	0.6
Urology infection + pneumonia + blood flow infection	3	0.9
Urology infection + pneumonia + Infectious endocarditis	1	0.3
Blood flow infection + spinal tuberculosis	1	0.3
Urology infection + spinal tuberculosis	1	0.3
** Unlocalized infection**	**57**	**17.8**
**Non-infectious inflammatory diseases**	**20**	**6.3**
** Autoimmune Diseases**	**12**	**3.8**
Systemic lupus erythematosus	6	
Rheumatoid arthritis	2	
Overlap syndrome	1	
Drying's Syndrome	1	
Reactive arthritis	1	
Undifferentiated connective tissue disease	1	
** Self-inflammatory disease**	**8**	**2.5**
Hemphage Syndrome	4	
Ulcerative colitis	2	
Adult still Disease	2	
**Tumor Diseases**	**16**	**5.0**
** Blood System Malignant Diseases**	**13**	**4.0**
Lymphomas	11	
Acute myeloid leukemia	2	
** Solid organ tumor**	**3**	**1.0**
Liver cancer	1	
Lung cancer	1	
Adrenal carcinoma	1	
**Other diseases**	**17**	**5.3**
Necrotizing lymphadenitis	8	
Subacute thyroiditis	8	
Drug Heat	1	
**Multiple causes**	**4**	**1.3**
Pneumonia + oesophageal cancer	1	
Pneumonia + subacute thyroiditis	1	
Urology infection + necrotizing lymphadenitis	1	
Viral meningitis + necrotizing lymphadenitis	1	
**Not diagnosed**	**41**	**12.8**

**Table 3:** Distribution of FUO etiology according to mean follow-up time(months)

	1 month (cases)	3 months (cases)	6 months (cases)	12 months (cases)	24 months (cases)	42 months (cases)
Infectious Diseases	40	—	41	52	53	36
NIID	6	—	—	2	8	4
Tumor Diseases	6	4	4	—	2	—
Other diseases	4	—	1	—	6	6
Multiple causes	1	—	—	2	1	—
Not diagnosed	13	—	4	8	7	9

In this paper, we also investigated differences in the etiological distribution of FUO in different years, genders, ages, and fever duration. From 2018 to 2019, the proportion of infectious diseases in FUO was higher than that from 2016 to 2017 (χ2 = 6.384, *P* = 0.012); the proportion of infectious diseases in FUO was higher in males and the elderly than in females and young and middle-aged (χ2 = 3.877, *P* = 0.049; χ2 = 8.543, *P* = 0.003; χ2 = 6.795, *P* = 0.009); And the proportion of diagnosed diseases was higher in young adults than in the elderly (χ2 = 10.035, *P* = 0.002). There was no statistically significant difference in the etiological distribution of FUO with different heat duration (*P* > 0.05). (See Table S1. See Additional file 1).

### Pathogen distribution

Among infectious diseases, bacterial infections were the most common (*n* = 185, 83.3%). Viral infection was found in 14 patients (6.3%), fungal infection in 1 patient (0.5%), and unknown pathogen in 22 patients (9.9%).Of the bacterial infections, 159 (71.6%) were bacteria other than Mycobacterium tuberculosis and Mycobacterium brucei, 24 (10.8%) were special bacteria, and 2 (0.9%) were mixed infections with multiple bacteria. There were a total of 27 cases of contagious diseases, accounting for 12.2% of infectious diseases and 8.4% of all causes, including 14 cases of Brucella, 11 cases of tuberculosis (including 2 cases with bacterial infection other than Mycobacterium tuberculosis and Mycobacterium brucei infection), 1 case of Salmonella typhi, and 1 case of hantavirus. (See Table 4).

**Table 4:** Pathogen distribution of infectious diseases

Pathogen	Number of examples	Proportion (%)
Bacteria infection	185	83.3
Bacteria other than Mycobacterium tuberculosis and Mycobacterium brucei	159	71.6
Special bacteria	24	10.8
Tuberculi	9	
Pulmonary Tuberculosis	1	
Expulmonary tuberculosis	8	
Brucelella	14	
Salmonella typhtyphoid	1	
Multiple bacterial mixed infections	2	0.9
Blood flow infection + spinal tuberculosis	1	
Urology infection + spinal tuberculosis	1	
Virus infection	14	6.3
EB virus	9	
Human Cytomegalo Virus(HCV)	2	
Hantavirus	1	
Herpes simplex virus(HSV)	1	
EB virus + Human Cytomegalo Virus(HCV)	1	
Fungal infection	1	0.5
Invasive pulmonary aspergillylosis	1	
Pathogen Unclear	22	9.9

### Follow-up and clinical outcome

**Fig. 2:**
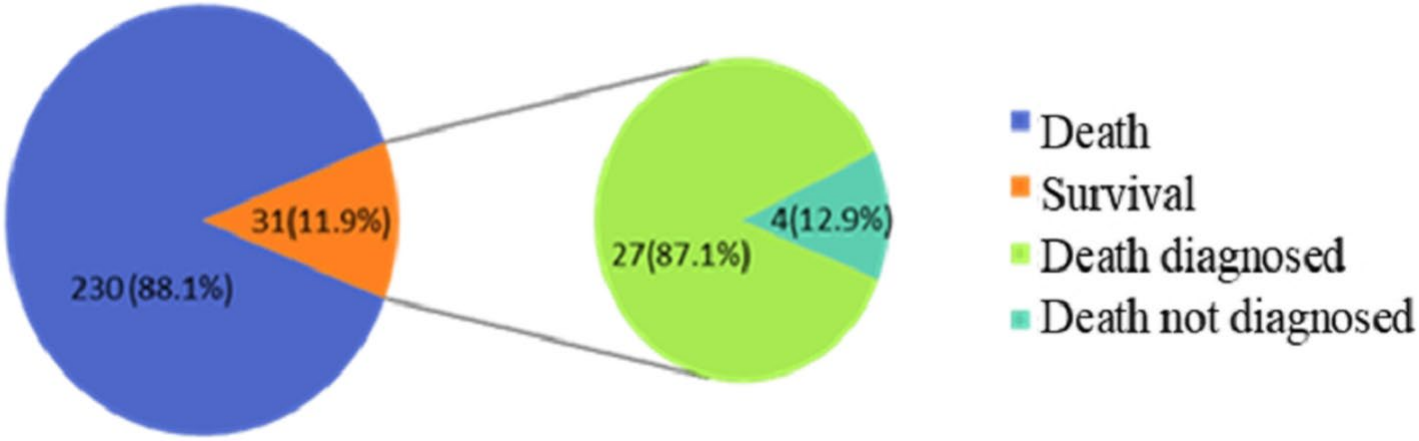
Follow-up results of 261 FUO patients

Of the 320 patients with FUO, 6 patients died during hospitalization (1.9%). Three hundred and fourteen patients were discharged, of whom 53 (16.9%) patients were lost to follow-up and 261 (83.1%) patients were with success followed up. 31 (11.9%) patients died and 230 (88.1%) patients survived. There were 230 (88.1%) patients diagnosed cases, 31 (11.9%) patients undiagnosed cases, 27 (11.7%) patients deaths among diagnosed cases, and 4 (12.9%) patients deaths among undiagnosed cases. (See Fig. 2.)Among the 41 undiagnosed follow-up patients in this study, 27 patients survived, 23 of whom had no further fever after 1 week of discharge, 1 patient whose fever resolved after 1 week of oral Chinese medicine, and 3 other patients who took other fever-reducing drugs intermittently and whose fever resolved at 0.5, 6, and 8 months, respectively.

Among the 6 patients who died during hospitalization, 5 patients were diagnosed; Three(60%) patients were male and two(40%)were female. 2 (40%) patients were young and 3 (60%) patients were middle-aged; The primary diseases were lymphoma in 1 patient, urinary tract infection with pneumonia in 1 patient, chronic active Epstein-Barr virus infection in 1 patient, and hemophagocytic syndrome in 2 patients; the direct causes of death were heart failure in 2 patients, respiratory failure in 1 patient, septic shock in 1 patient, and upper gastrointestinal bleeding in 1 patient. A 36-year-old woman presented with intermittent fever of unknown etiology and eventually died due to cardio-respiratory arrest (See Table 5).

**Table 5:** Characteristics of Patients with Death in FUO

	Gender	Age (year of age)	Follow-up time (month)	Primary disease	Direct cause of death
**Death during the hospitalization** **(6 Patients)**					
Diagnosis (5 Patients)	Male	55	—	Lymphomas	Respiratory failure
	Female	59	—	Urology system infection + pneumonia	Infectious shock
	Male	40	—	Hemphage Syndrome	Heart failure
	Male	45	—	Hemphage Syndrome	Heart failure
	Female	39	—	Chronic Active EB Virus infection	Upper gastrointestinal bleeding
Not diagnosed (1 Patient)	Female	36	—	Unknown	Cardiac respiratory arrest
**Death during the follow-up period** **(31 Patients)**					
Diagnosis (27 Patients)					
Male		65	24	Acute myeloid leukemia	Intracerebral hemorrhage
Male		67	3	Acute myeloid leukemia	Infectious shock
Female		48	6	Lymphomas	Respiratory failure
Female		69	5	Lymphomas	Heart failure
Female		17	1	Lymphomas	Infectious shock
Female		70	1	Lymphomas	Respiratory failure
Male		15	3	Lymphomas	Infectious shock
Female		70	3	Lymphomas	Respiratory failure
Male		62	4	Lymphomas	Intracerebral hemorrhage
Male		60	12	Blood flow infection	Infectious shock
Female		75	24	Spinal tuberculosis	Intracerebral hemorrhage
Male		84	12	Urinary Infection	Infectious shock
Female		66	11	Urinary Infection	Brain infarction
Male		71	12	Cholecystitis	Infectious shock
Male		75	2	Urinary Infection + pneumonia	Myocardial infarction
Female		63	1	Hemphage Syndrome	Heart failure
Male		73	16	Urology infection + pneumonia + blood flow infection	Infectious shock
Male		83	6	Urology system infection + pneumonia	Infectious shock
Male		39	3	Infectious endocarditis	Heart failure
Male		39	1	Infectious endocarditis + pneumonia + urinary infection	Infectious shock
Male		62	7	Infectious endocarditis	Myocardial infarction
Female		71	8	Adrenal abscess	Myocardial infarction
Male		48	8	Infectious endocarditis, + pneumonia	Heart failure
Female		68	6	Skin soft-tissue infection + pneumonia	Myocardial infarction
Female		56	5	Lung cancer	Respiratory failure
Female		69	6	Liver cancer	Liver failure
Female		60	5	Esophageal Cancer	Upper gastrointestinal bleeding
**Not diagnosed (4 Patients)**					
Female		53	12	Unknown	Intracerebral hemorrhage
Female		51	1	Unknown	Respiratory failure
Male		82	2	Unknown	Myocardial infarction
Male		60	1	Unknown	Unknown cause

Among the 31 patients who died during the follow-up period, 27 patients were diagnosed; 14(51.9%) patients were male and 13(48.1%) were female. Four patients (14.8%) were young adults, three were middle-aged adults (11.1%), and 20 were elderly (74.1%).The primary diseases included infectious diseases in 14 patients, neoplastic diseases in 12 patients, and non-infectious inflammatory diseases in 1 patient; The direct causes of death were 9 patients of infectious shock, 3 patients of cerebral hemorrhage, 4 patients of respiratory failure, 4 patients of heart failure, 4 patients of myocardial infarction, 1 patient of cerebral infarction, 1 patient of liver failure, and 1 patient of upper gastrointestinal hemorrhage. There were four deaths of undiagnosed patients, two in each sex, two in middle age and two in old age. One patient still had intermittent fever after discharge, and the cause of death was unknown, the other 3 patients had no fever after discharge and died of myocardial infarction, cerebral hemorrhage, and respiratory failure, respectively (See Table 5).

## Discussion

The etiology of fever of unknown origin is complex, and the etiology is influenced by time, region and other factors, and the etiology of FUO is closely related to the prognosis [4–7]. Therefore, dynamic study of the etiology and prognosis of FUO is of great benefit to improve the level of clinical diagnosis and treatment. We applied a structured diagnostic protocol to prospectively study the etiology and prognosis of FUO and found that infectious diseases were the primary cause of FUO, urinary system and lung were the two most common sites of infection, and the pathogens were mainly bacteria other than Mycobacterium tuberculosis and Mycobacterium brucei. This is followed by non-infectious inflammatory diseases, other diseases, neoplastic diseases, and multiple etiologies. Brucellosis is the most common contagious disease. Patients with FUO have a low in-hospital mortality rate, deaths after discharge are mostly related to the primary disease, and undiagnosed patients mostly die of cardiovascular and cerebrovascular diseases. In this study, the confirmed rate of FUO was 87.2%, and infectious diseases were the primary cause, which was similar to the results of Zhai Pan and Wang Yuqiang [22, 23]. Notably, the etiology of FUO varies between countries (see Table 6).

**Table 6:** Etiological composition of FUO in the literature

Year(Ref)	Country	Study type	Total number	ID (%)	MD (%)	NIID (%)	Mis (%)	U (%)
1961 [1]	America	P	100	36.0	19.0	19.0	19.0	7.0
2003 [20]	Belgium	P	185	10.8	9.7	18.4	8.1	53.0
2006 [24]	Jordan	R	52	50.0	15.0	12.0	8.0	15.0
2007 [25]	Netherlands	P	73	16.0	7.0	22.0	4.0	51.0
2010 [26]	Greece	P	112	30.4	10.7	33.0	5.4	20.5
2012 [27]	Denmark	R	52	19.2	7.7	32.7	0	30.4
2013 [28]	China	R	997	48.0	7.9	16.9	7.1	20.1
2014 [29]	India	P	91	44.0	12.1	12.1	4.4	27.4
2016 [30]	Bulgaria	R	54	59.3	3.7	14.8	5.5	16.7
2017 [31]	Japan	R	42	17.0	12.0	43.0	7.0	21.0
2017 [32]	Netherlands	R	236	16.1	6.8	31.4	5.1	40.7
2018 [33]	Iran	R	101	23.1	17.9	21.1	5.3	32.6
2019 [7]	Japan	P	141	17.0	15.6	34.0	12.1	21.3
2020 [34]	China	R	1641	48.69	16.94	19.26	6.76	8.35
2020 [9]	India	P + R	152	43.4	21.5	19.7	2	12.5
2021 [12]	Turkey	R	214	44.9	15.42	11.68	8.41	19.62

*P* Prospective, *R* Retrospective, *ID* Infectious diseases, *MD* Malignant diseases, *NIID* Non-infectious inflammatory diseases, *Mis* Miscellaneous diseases, *U* Undiagnosed

However, as reported by Takeda R etc., in developed countries, non-infectious inflammatory diseases have gradually increased and become the main cause of FUO, accounting for 22% to 43% [25, 27, 31]. The above differences in etiological distribution may be related to public health facilities, diagnostic techniques, and level of economic development [5, 6].

In this study,the urinary system was found to be the most common site of infection. Most FUO patients with urinary tract infection only present with fever and lack urinary tract irritation symptoms such as frequent urination, urgency, and dysuria, so imaging examination is an important means of determining special types of urinary tract infection, and 8 patients of acute focal bacterial nephritis(AFBN) in this study were diagnosed by abdominal enhanced CT. Acute focal bacterial nephritis is a rare localized bacterial infection of the renal interstitium that can occur at all ages, but is more common in children and the diagnosis is hysteretic due to the lack of specific symptoms. Antibiotics are the mainstay of treatment for this disease, and most studies consider appropriate courses of treatment to be 2–4 weeks [35–37], however, up to 6 weeks in individual patients [21]. All 8 patients in this study received a period of empirical anti-infective treatment at other hospital, but due to the long course of antibiotics for AFBN and poor effect of a short course of treatment, they were transferred to our department with fever of unknown origin, and then the diagnosis was confirmed by intensive CT examination. This suggests that for FUO patients who cannot be diagnosed by general examination, enhanced abdominal CT can be perfected to further clarify the presence of insidious urinary tract infection. The lung is the next most common site of infection. The majority of patients with lung infection caused by FUO had no conventional respiratory symptoms such as cough or sputum, and the majority of them had a chest X-ray prior to admission.However, chest CT has higher resolution and diagnostic value compared with chest X-ray. Bleeker-Rovers et al. [25] showed that chest radiography sensitivity was only 60%, while chest CT reached 82%. Therefore, routine chest CT in FUO patients is extremely recommended.

The most common contagious disease in this investigation was Brucellosis. Brucellosis as the most common infectious disease in FUO may be associated with the following factors. First, brucellosis has diverse clinical manifestations [38] and early diagnosis is difficult. In this study, there were 14 patients of brucellosis, of which only 3 patients showed common manifestations of brucellosis infection such as muscle soreness and arthralgia, and the remaining patients only showed fever. Second, although blood culture is an important method to confirm Brucella infection, sensitivity is influenced by many factors [39]. In addition, insufficient awareness of brucellosis by clinicians is also an important factor. All brucellosis patients in this study were treated with antibiotics before admission, but the diagnosis was not confirmed early limited to the physician 's level of awareness, and similar conditions occurred in other countries [12, 40].

NIID are the second cause of FUO, the most common of which is systemic lupus erythematosus(SLE), which differs from related findings [9, 27, 41], and this difference may be caused by two factors. On the one hand, the age composition of the study population is an important factor affecting the etiological distribution of FUO. A Japanese multicenter study by Naito T et al. [42] included FUO patients with a median age of 59 (19 ~ 94) years, and polymyalgia rheumatica emerged as the most common cause due to the aging population. On the other hand, differences in disease incidence between regions may also have some impact. A systematic review by Rees et al. [43] showed that the incidence of SLE varies worldwide by gender, age, race, and Period.

Histiocytic necrotizing lymphadenitis(HNL), Also called Kikuchi – Fujimoto disease(KFD), and subacute thyroiditis(SAT) were the common types of other diseases in this study. HNL is a rare disease characterized by regional lymph node necrosis with diverse clinical manifestations [44] and is easily misdiagnosed as other benign lymphadenopathies or lymphomas [45]. The diagnosis of HNL depends on pathological examination of a certain amount of lymph nodes taken by open surgery [44, 45], but some patients refuse the examination at the early stage of the disease so that they cannot be diagnosed early. Subacute thyroiditis(SAT) is not a common cause of FUO, and the diagnosis requires a comprehensive analysis of symptoms, signs, thyroid function tests, and thyroid radionuclide scan results. Typical symptoms such as neck and pharyngeal pain are present in a low proportion of FUO associated with SAT and are difficult to diagnose. In our study, only 25% of SAT patients with neck pain, 25% developed sore throat, and the rest had no symptoms except fever. A Polish study showed an increase in SAT without pain compared with the past [46]. In addition, patients' concerns about radioactivity from radionuclide examinations may also be important factors.

Neoplastic diseases are one of the important causes of FUO, of which lymphoma is the main cause. Due to the application of imaging and serological tumor markers, solid tumors can be diagnosed early and account for a decrease in FUO; hematologic tumors are difficult to diagnose and the proportion in tumor-related FUO gradually increases, especially lymphoma [9, 34, 47]. The clinical manifestations of lymphoma vary, and histopathological examination is an important diagnostic method. It is necessary to perform additional biopsies in difficult cases, especially when malignant diseases are suspected, in order to improve the diagnosis rate [13]. In our study, 90.9% (10/11) of lymphoma patients presented with non-specific symptoms such as fever and fatigue, and only one patient developed lymphadenopathy. Of the 11 patients, 3 were diagnosed by paranasal sinus, gastric, and skin biopsies, respectively;1 patient with lymphadenopathy showed non-specific inflammatory changes on the first inguinal lymph node biopsy, which was later diagnosed as Hodgkin lymphoma by bone marrow aspiration; the remaining patients were diagnosed by bone marrow aspiration. The possible reasons for the difficulty in diagnosing lymphoma patients with FUO as the first manifestation are as follows: ① A small number of patients require multiple invasive examinations to confirm the diagnosis [13]; ②Irregular fever of lymphoma lasts for a long time [46].

Related studies have shown a decreasing trend in the proportion of FUO associated with infection over time [47, 48]. However, this study found an increase in the proportion of infectious diseases from 2018 to 2019 compared to 2016 to 2017. The reason why this study differs from other related study results may be different study methods and study duration. In addition, the development of diagnostic techniques may also play a role. With the application of second-generation DNA sequencing and other technologies in clinical practice, the diagnostic ability of infectious diseases has been further improved [49–51], so that infectious diseases that could not be diagnosed in the past have been diagnosed, and then the proportion of infection-related FUO has increased. Therefore, along with the changes in diagnostic techniques, dynamic study of the etiological changes of FUO is essential to improve the level of FUO understanding.

A Serbian study showed [52] that the prevalence of rheumatism was high among female, elderly FUO patients, while the prevalence of infectious diseases was high among male, young and middle-aged FUO patients. Jia Weihua 's study found [16] that although there was no difference in the etiological distribution of FUO between genders, elderly FUO patients were more susceptible to infectious diseases than young and middle-aged adults, and at the same time, the proportion of non-infectious diseases was higher in young and middle-aged adults. In this study, we found that the proportion of infectious diseases in FUO was higher in men and the elderly than in women and young and middle-aged adults, and the proportion of undiagnosed diseases was higher in young adults than in the elderly, which was different from the results of other studies.

Kabapy et al. [53] retrospectively studied the prognosis of 979 FUO patients, 2.2% of whom died during hospitalization. The in-hospital mortality rate of FUO patients in our study was 1.9%, which was similar to the relevant findings [53]. In our study, the patients who died during hospitalization were mainly diagnosed cases, 60% were middle-aged patients, and heart failure was the most common direct cause of death. This suggests that we should be alert to the risk of cardiac mortality in middle-aged FUO patients diagnosed during hospitalization. Vanderschueren et al. [54] followed 436 patients with FUO in Belgium for at least 6 months and the mortality rate during follow-up was 10.1%. A retrospective study by Chinese scholar Ji Weijia et al. [8] showed that the mortality rate of 1838 FUO patients was only 0.38% during a 3-month follow-up. In this study, the survival rate of the patients followed was 88.1% and the mortality rate was 11.9%. The different mortality rates of FUO patients between studies may be related to different follow-up time, age of included patients and disease composition During the follow-up period, the mortality rate of diagnosed cases in this study was 11.7%, 85.2% of middle-aged and elderly patients, the primary disease was mainly infectious diseases, and septic shock was the first direct cause of death. In the study by Ji Weijia et al. [8], 57.1% of patients died of sepsis, and our study was consistent with it. However, in the study by Vanderschueren et al. [54],60% of patients died of neoplastic disease. The above differences may be related to the different etiological distribution of FUO between regions. The mortality rate of patients who were not diagnosed during the follow-up period in this study was 12.9%. Li Yuanjie et al. [15] conducted a follow-up study of FUO patients who were not diagnosed at discharge for a median of 76 months, and the mortality rate of patients who were finally not diagnosed was 27.8%, which was higher than that of our study and may be related to the fact that the follow-up time of the study was significantly longer than that of our follow-up time. Age is associated with the prognosis of FUO patients [54], and patients who did not have a diagnosis of death in this study were all middle-aged and elderly, with cardiovascular and cerebrovascular diseases accounting for 50% of the direct causes of death. This suggests that patients with undiagnosed FUO are also at risk of death and mostly die of cardiovascular and cerebrovascular diseases, and follow-up monitoring should be strengthened.

In this study, a multidisciplinary expert FUO research team was established to develop a structured diagnostic protocol, prospectively applied to the etiological study of FUO, and follow up its prognosis. Currently, the outcomes of patients discharged with undiagnosed FUO have been reported in only a few studies. The longer follow-up period of our study enabled us to obtain more accurate information due to the larger sample size.

There are some limitations in this study.First,because of many differences in management and diagnostic facilities exist among hospitals or countries. The use of the non-validated diagnostic scheme protocol has some limitations. Second, serum electrophoresis was not performed in the patients in this study. Third, FDG-PET has only been done in a few cases, due to the patient's financial considerations.It could be applied to more patients with FUO in the future.Last, follow-up of undiagnosed cases in this study was not robust.

## Conclutions

In developing countries, the primary cause of FUO is infectious diseases. There are temporal differences in the etiological distribution of FUO, and the etiological distribution of FUO patients who present in different years is different, and the etiology of FUO is closely related to the prognosis. Along with the changes of diagnostic techniques, dynamic study of the etiological changes of FUO is essential to improve the level of FUO understanding. FUO patients have a low in-hospital mortality rate, deaths after discharge are mostly related to the primary disease, and undiagnosed patients have a high mortality rate, so it is very important to identify the etiology of patients with worsening or unremitted disease.

## Supplementary Information

**Additional file 1.**

## Data Availability

All data analysed during this study are available from the corresponding author on reasonable request.

## Abbreviations

FUO: Fever of Unknown Origin
NIID: Non-infectious Inflammatory Diseases
CT: Computed tomography
18F-FDG PET/CT: 2-[18F]-fluoro-2-deoxy-D-glucose—Positron Emission Tomography/Computed Tomography
NK cell: Naturalkiller cell
SLE: Systemic Lupus Erythematosus
HCV: Human Cytomegalo Virus
HSV: Herpes Simplex Virus
AFBN: Acute Focal Bacterial Nephritis
HNL: Histiocytic necrotizing lymphadenitis
KFD: Kikuchi – Fujimoto disease
SAT: Subacute Thyroiditis
