# Supplementary material for: Structured diagnostic scheme clinical experience sharing: a prospective study of 320 cases of fever of unknown origin in a tertiary hospital in North China
Source: BMC Infect Dis. 2023 Jul 7;23:452. doi: 10.1186/s12879-023-08436-0 (PMC10327337; doi:10.1186/s12879-023-08436-0)
Supplement: Supplementary file 1 — Additional file 1. [file 12879_2023_8436_MOESM1_ESM.docx]

| Causes | Year: | |  | Gender | |  | Age (year of age) | | |  | Heat range (month) | | |
| --- | --- | --- | --- | --- | --- | --- | --- | --- | --- | --- | --- | --- | --- |
|  | 2016-2017 (n=96) | 2018-2019 (n=220) |  | Male (n=158) | Female  (n=158) |  | ≤ 44 (n=109) | 45-59  (n=84) | ≥ 60 (n=123) |  | ＜1  (n=95) | 1-3  (n=168) | ＞3  (n=53) |
| Infectious Diseases | 58（60.4） | 164（74.5）* |  | 119（75.3） | 103  （65.2）▲ |  | 69  （63.3） | 54  （64.3） | 99  （80.5）# |  | 69  （72.6） | 117  （69.6） | 36  （67.9） |
| Noninfectious diseases | 22（22.9） | 31（14.1） |  | 21  （13.3） | 32  （20.3） |  | 19  （17.4） | 17  （20.2） | 17  （13.8） |  | 18  （18.9） | 29  （17.3） | 6  （11.3） |
| Not diagnosed | 16（16.7） | 25（11.4） |  | 18  （11.4） | 23  （14.6） |  | 21  （19.3） | 13  （15.5） | 7  （5.7）★ |  | 8（8.4） | 22  （13.1） | 11  （20.8） |

**Table S1**. Differences in Cause Distribution of FUO in Different Years, Sex, Age, and Heat Range [Example (%)]

* P <0.05, vs 2016-2017; ▲ P <0.05, vs male; # P <0.01, vs 45-59 years old and less than or equal to 44 years old;

★ P <0.01, vs less than or equal to 44 years old.
